# What is the importance of solitary focal bony FDG-uptake on 18F-FDG PET-CT of known cancer patients?

**DOI:** 10.1186/1470-7330-14-S1-P6

**Published:** 2014-10-09

**Authors:** LI Sonoda, A Lakhani, S Ghosh-Ray

**Affiliations:** 1 Paul Strickland Scanner Centre, Mount Vernon Hospital, London, UK

## Aim

18F-FDG-PET-CT plays an important role in oncology staging. While the presence of multiple FDG-avid lesions on PET-CT in the context of known malignancy is generally considered metastases, the exact significance of solitary FDG-avid-lesions remains unknown. This study was undertaken to evaluate the significance of solitary bony lesions on PET-CT of oncology patients.

## Methods

Retrospective review of 15,645 PET-CT studies was performed. Further evaluation of solitary bony FDG-avid lesions was carried out by conventional imaging, follow-up and biopsy studies. Spontaneous resolution on subsequent PET-CT without a change in therapy was considered benign while progression was considered malignant.

## Results

361 (3%) cases were found to have single FDG-avid skeletal lesions, of which 16 were due to uptake at the primary bony malignancy, and 42 were not further-investigated/passed away, hence excluded. Of the remaining 303 lesions 276 (91%) were confirmed as metastases, 27 (9%) proven benign (10 by imaging, 5 by biopsy and12 by follow-up).

Of 276 metastases (SUVmax 9.6+/-6.6); 191 were lytic, 45 sclerotic, 21 mixed and 19 normal on CT. Of 27 benign (SUVmax 3.8+/-2.8); 2 were lytic, 7 sclerotic, 2 mixed and 16 normal on CT. PPV of PET-CT on lytic, sclerotic, mixed and normal lesions on CT are 99%, 87%, 91% and 54% respectively. There was significant difference in SUVmax between malignant/benign lesions (P<0.001).

PET-CT correctly upstaged in 83/303 (27%) cases, but incorrectly upstaged or suggested further investigation in 18/303 (6%) cases.

## Conclusion

Solitary skeletal FDG-uptake on 18F-FDG-PET-CT in patients with known malignancy is just as significant as multiple skeletal FDG-uptake, carrying high risk of metastases.

